# A novel group A rotavirus associated with acute illness and hepatic necrosis in pigeons (*Columba livia*), in Australia

**DOI:** 10.1371/journal.pone.0203853

**Published:** 2018-09-11

**Authors:** Christina McCowan, Sandra Crameri, Ayfer Kocak, Songhua Shan, Mark Fegan, David Forshaw, Dennis Rubbenstroth, Honglei Chen, Clare Holmes, Jenni Harper, Megan Dearnley, Jana Batovska, Jemma Bergfeld, Colin Walker, Jianning Wang

**Affiliations:** 1 Agriculture Victoria, Bundoora, Victoria, Australia; 2 Australian Animal Health Laboratory, Geelong, Victoria, Australia; 3 Department of Primary Industries and Regional Development, Albany, Western Australia, Australia; 4 Institute of Virology, Medical Center–University of Freiburg, Freiburg, Germany; 5 Institute for Diagnostic Virology, Friedrich-Loeffler-Institute (FLI), Greifswald–Insel Riems, Germany; 6 School of Applied Systems Biology, La Trobe University, Bundoora, Victoria, Australia; 7 Melbourne Bird Veterinary Clinic, Scoresby, Melbourne, Australia; Tulane University, UNITED STATES

## Abstract

Cases of vomiting and diarrhoea were reported in racing pigeons in Western Australia in May, 2016. Morbidity and mortality rates were high. Similar clinical disease was seen in Victoria in December and by early 2017 had been reported in all states except the Northern Territory, in different classes of domestic pigeon–racing, fancy and meat bird–and in a flock of feral pigeons. Autopsy findings were frequently unremarkable; histological examination demonstrated significant hepatic necrosis as the major and consistent lesion, often with minimal inflammatory infiltration. Negative contrast tissue suspension and thin section transmission electron microscopy of liver demonstrated virus particles consistent with a member of the *Reoviridae*. Inoculation of trypsin-treated Vero, MDBK and MA-104 cell lines resulted in cytopathic changes at two days after infection. Next generation sequencing was undertaken using fresh liver samples and a previously undescribed group A rotavirus (genotype G18P[17]) of avian origin was identified and the virus was isolated in several cell lines. A q-RT-PCR assay was developed and used to screen a wider range of samples, including recovered birds. Episodes of disease have continued to occur and to reoccur in previously recovered lofts, with variable virulence reported. This is the first report of a rotavirus associated with hepatic necrosis in any avian species.

## Introduction

Avian rotaviruses were first detected in turkeys with diarrhoea, in the USA in 1977 and, the following year, in the UK [1]. Experimental infection has shown that chickens less than 56 days old are likely to be subclinically infected [2–4], while turkeys and older chickens may develop diarrhoea [4]. Exposure at a young age may protect older chickens from overt disease. Domestic poultry flocks have been shown to harbour rotaviruses, with prevalence ranging from 18.8% to 69.7% of turkey flocks and 9.9% to 46.5% of chicken flocks worldwide [5–8]. In most surveys both clinically affected and clinically unaffected birds were found to be shedding rotavirus RNA in faeces, so the significance of this is uncertain. Simultaneous or sequential infections with different rotaviral groups is frequently observed in broiler chicken and turkey flocks [1, 9–11].

A wide range of avian species has since been shown to shed rotaviruses, including mallard ducks, reed buntings, pheasants, quail and guinea fowl [12–14] and, in contrast to domestic poultry, disease in most species may be more common in birds under six weeks of age than in adults [1]. Rotaviruses in wild and feral birds are generally found at low levels [14]. In feral pigeons, serological evidence of rotavirus A infection has ranged from 10.7% - 68% in various locations [15–17]. The PO-13 strain was isolated from a feral pigeon and subsequently shown to infect mammalian cells *in vitro* [17]. This strain was the first avian rotavirus genome to be fully sequenced [18], and provided the first experimental evidence of avian to mammalian cross species pathogenesis [19]. Avian-like group A rotaviruses are reported to be associated with natural mammalian infections [20–22]. In pheasants, turkeys and chickens there have been reports of rotaviruses containing both avian and mammalian genomic sequences being isolated [23–25].

In mammals, rotaviruses are generally recognised to be gastroenteric pathogens of neonates [26] although the pathogenesis of disease is incompletely understood [27]. However, there is increasing recognition that infection may be associated with viraemia [27–32], and disease other than enteritis has been reported in both humans and animals [20, 27, 33] although not yet in birds.

We investigated vomiting and diarrhoea associated with high mortality in domestic pigeons (*Columba livia*). report the isolation and characterisation of a previously undescribed rotavirus associated with intestinal signs and hepatic necrosis in domestic and feral pigeons (*Columba livia*). The disease epidemiology suggested an infectious agent and histological findings were suggestive of a viral infection, but routine bacterial and viral screening were unrewarding. Using electron microscopy (EM), virus isolation and molecular techniques including next generation sequencing (NGS) we identified a previously undescribed rotavirus associated with intestinal signs and hepatic necrosis.

A qRT-PCR assay was developed for use in diagnostic testing and surveillance. This is the first report of extra-intestinal rotavirus infection in an avian species.

## Materials and methods

### History of disease

In 2016 and 2017, pigeons were submitted moribund or dead for autopsy, or fresh and formalin fixed tissue samples or cloacal swabs were submitted from a referring private avian practice to the state government laboratories in Western Australia (DPIRD) or Victoria (DEDJTR). Further samples were referred to the DEDJTR or to CSIRO AAHL from other states. Where possible, flock and individual clinical information associated with each submission were collected and collated.

### Gross and microscopic pathology

Moribund birds were euthanased with CO_2_ or intravenous barbiturate. At autopsy, cloacal and choanal swabs for routine exclusion of avian influenza virus, Newcastle disease virus and pigeon paramyxovirus were placed in viral transport medium (VTM). Fresh samples, including liver, were taken for routine bacteriology and negative contrast electron microscopy (EM). Fresh tissue was also collected in VTM; initially pooled liver, kidney, spleen and pancreas were taken. At later autopsies, following the histological results, only liver was collected.

Tissues including viscera, muscle, peripheral and central nervous systems and eyes were placed in 10% neutral buffered formalin. Liver from three birds and spleen from one were fixed in 2.5% glutaraldehyde for transmission EM.

Formalin fixed tissues were processed and cut, and sections stained with haematoxylin and eosin in routine fashion for histological examination.

### Pathogen screening

Liver and faeces from three Western Australian birds, a further seven livers only and 19 livers from Victorian birds were submitted for bacterial culture. For aerobic culture, all samples were streaked onto sheep blood and McConkey agars. All Victorian samples and six Western Australian samples, including one faecal sample, were enriched in selenite broth and streaked onto BLXDG agar for *Salmonella* sp. isolation. Incubation was performed at 37°C.

For anaerobic culture, two Western Australian and two Victorian liver samples were streaked onto sheep blood agar and incubated at 37C under anaerobic conditions.

Four splenic samples from Western Australian birds and one of liver from Victoria were subjected to DNA extraction and PCR amplification for generic identification of *Chlamydia* sp.

Three splenic samples and one swab each from cloaca and choana, all from Western Australian birds, were submitted for generic herpesvirus PCR. Samples from four Western Australian birds were sent to an external provider for generic adenovirus PCR.

### Electron microscopy

For negative contrast EM, liver tissue was homogenised in Phosphate Buffered Saline (PBS) 20% w/v and centrifuged at 17,000 x g for 5 minutes. The supernatant was adsorbed onto carbon-coated formvar copper grids and stained with nanoW (Nanoprobes, NY, USA) for 1 min. Cell culture media from virus isolation attempts, clarified by centrifugation (as above), were similarly prepared.

For thin section EM, glutaraldehyde fixed liver tissue or cell pellets from virus isolation attempts, were postfixed with 1% osmium tetroxide for 1 hour and embedded in Spurr’s resin (ProSciTech, QLD, Aus) in routine fashion. Ultrathin sections were stained with saturated uranyl acetate in 50% ethanol followed by lead citrate. All prepared grids were examined using a Philips CM120 transmission electron microscope at 120kV.

### Virus isolation

#### Sample preparation

Mixed tissues or fresh liver only, were submitted in 1ml VTM. The samples were activated by adding 5 to 10 μg/ml trypsin followed by incubation for 30 minutes at 37°C to increase rotavirus infectivity [17, 34].

#### Cell cultures and media

African green monkey kidney cell lines (MA104, Vero), Madin-Darby bovine kidney cell line (MDBK) and chicken liver hepatocellular carcinoma cell line (LMH) were purchased from ATCC. Chicken embryo liver (CEL) cells were prepared from 14 to 16 –day-old SPF chicken embryos [35]

Cells were cultivated in tissue culture flasks (T25) (Corning, Inc., Cat. No.002019). to 95–100% confluency in a growth medium (GM) containing 7 to 10% Foetal Bovine Serum (FBS), 100 U/ml of penicillin, and 100 μg/ml of streptomycin. After virus inoculation the cells were maintained in a maintenance medium with either 0.5% FBS (MM) or replaced by 0.5–1.0 μg/ml trypsin (Sigma Cat. No.59427C) without FBS (MMT). All cells were incubated at 37°C and 5% CO_2_.

#### Virus isolation, identification and quantification

After removal of GM, undiluted or 10-fold to 1,000-fold diluted activated samples were added to cell culture flasks. Uninoculated cells and cells inoculated with trypsin-supplemented VTM alone served as controls. After adsorption for one hour at 37°C the inoculum was removed and the cells were incubated in MM or MMT for up to seven days observing for signs of cytopathic effect (CPE). Thereafter cultures were subjected to further passages following two to three freeze/thaw cycles at -80°C and then the process of trypsinisation and inoculation.

Following observation of CPE, confirmation of virus isolation was made using PCR, EM or immunofluorescence assay (IFA).

#### Immunofluorescence assay (IFA)

Fluorescent labelling was performed using standard techniques. Twenty-four-well MA104 cell culture plates were seeded with 4 x 10^4^ cells per well and infected with 10-fold serially diluted rotavirus preparations in the presence of trypsin. Goat anti-rotavirus antibody (1:100, Millipore, Cat. No. AB1129) was applied at 37°C for 60 min and followed by donkey anti-goat IgG conjugate (1:200 Invitrogen™ Alexa Fluor 488 dye) with 0.005% Evans blue diluent (Koch-Light Laboratories Ltd, England, Cat. No. 0589–60) containing 1% BSA at 37°C for 30 min. Cell monolayers were observed under an Olympus IX83 inverted microscope.

### Next generation sequencing (NGS) analysis

#### Samples and nucleic acid extraction

Liver samples were collected at autopsy into VTM. For sequencing a small section of the liver sample was homogenised in fresh VTM and then centrifuged at 10,000 x *g* for three minutes to clarify the sample before supernatant was extracted with the QIAamp Viral RNA Mini Kit (Qiagen) according to the manufacturer’s instructions.

#### Construction and sequencing of metagenomic libraries

The RNA was processed using the NEBNext Ultra RNA Library Prep Kit for Illumina (New England Biolabs), according to the manufacturer’s instructions. Final libraries were quantitated on a Qubit Fluorometer and an Agilent Tapestation 2200 before normalising the libraries, and equimolar pooling. The library pool was then sequenced using a MiSeq sequencing reagent Version 3 (2 x 300 cycle) cartridge according to the manufacturer’s instructions, and loaded on a MiSeq machine.

#### NGS data analysis

The data were analysed using CLC Genomics Workbench v10.1 with standard parameters or a custom Perl script to trim sequences with a median Q score of <20, three or more nucleotides (nt) with a Q score of <20, three or more consecutive ambiguous bases or that were shorter than 100 nt. Illumina adaptors were removed from the reads using Cutadapt version 1.9 [36].

The trimmed reads were aligned to a pigeon reference sequence (*Columbia livia*, NCBI accession GCF_000337935.1) using BWA-MEM version 0.7.7 [37].

*De novo* assembly was then conducted with the unaligned sequence reads, to generate longer sequence contigs. The resultant sequences were then analysed using the NCBI nonredundant nucleotide database (BlastN) version 2.3.0 and protein database (BlastX).

#### Phylogeny

Phylogenetic comparison of VP7 gene from the detected genome with published rotaviral genomes, both avian and mammalian, was undertaken using MEGA software, version 7 [38], and a phylogenetic tree was generated using a Maximum Likelihood method.

### Development of quantitative PCR assays

Quantitative RT-PCR (qRT-PCR) assays were developed based on the rotavirus VP6, VP7, and NSP3 sequences obtained from NGS data. A MagMAX™-96 Viral RNA Isolation Kit (ThermoFisher) was used for RNA extraction from all sample types as per the manufacturers’ instructions. The sequences of primers and probes are summarised in Table 1. For real-time PCR an AgPath-ID One-Step RT-PCR master mix (ThermoFisher) was used on an ABI Prism® 7500 Fast Real-Time PCR System. RT-PCR was completed using a reaction volume of 25μL, each primer was used at a final concentration of 400nM and probes at a final concentration of 120nM. The cycling conditions for the qPCR assays were; 45˚C 10min, 95˚C 10min followed by 45 cycles of 95˚C for 15s and 60˚C for 40s.

**Table 1:** Primer sets tested for rotavirus detection.

Region	Primer/Probe	Sequence (5’-3’)
VP-6	Forward	GCCCGCAATTTCGATTCAATACG
VP-6	Reverse	GTGCTGCTACTCCAGGTGTCAT
VP-6	Probe	6FAM-TTCCAACTTGTTAGGCCGCCAA-BHQ
VP-7	Forward	GGGTGTCGGACAACTGATGTAG
VP-7	Reverse	TGCACGATGCGACTGTATAATTG
VP-7	Probe	6FAM-CATTCGAGCAGTTAACAACCGCTGA-BHQ
NSP3	Forward	GCAAAGATACGCTGCAAGATGA
NSP3	Reverse	TGACGCCATTCTCCCACTAAG
NSP3	Probe	6FAM-TGGATGATTCTGGTGTACAAGCTAACATGT-BHQ

A comparison of primer sets and sample types was made. For known positive samples, nucleic acid was extracted from fresh tissues and/or cloacal swabs taken from pigeons submitted for autopsy (n = 10) and demonstrated to have hepatic necrosis. Known negative samples were derived from swabs (location not specified) submitted with formalin fixed tissues taken at external autopsy of birds without characteristic hepatic necrosis and another cause of death (n = 5, including one feral pigeon and two native pigeons). Swabs submitted without tissues from birds with clinical suspicion of rotavirus associated disease (n = 22, including samples from a Queensland loft with low mortality) and cloacal and choanal swabs from birds from two clinically unaffected lofts (n = 20) were also tested, as were swabs taken from birds that had recovered from clinical disease at four (n = 4), seven (n = 10) and ten weeks (n = 11) following resolution of signs. Cloacal swabs were also received without further information on health status (n = 16). Positive and negative controls from cell culture were included. 120 samples were tested with all three primer sets. Further submissions were tested with the chosen screening assay, as were 92 archival samples retained from previous pigeon investigations predating this outbreak.

## Results

### History of disease

Between May 20 and June 21, 2016 disease was reported in eight racing pigeon lofts and one fancy pigeon loft in geographically dispersed locations in the metropolitan area of Perth, Western Australia. Epidemiological investigations were hampered by incomplete information provided by owners but in two lofts, 50 out of 250 and 25 out of 120 racing pigeons respectively were reported to have died at the time of presenting dead birds for examination. Birds in the affected lofts had been observed going light, regurgitating and developing diarrhoea, or simply found dead. Illness affected birds of both sexes and all ages. A flock of feral pigeons at a grain silo was anecdotally reported to have showed signs of disease with approximately 160 dead birds.

Birds from three affected lofts were reported to have shared a race transport vehicle three to four days prior to illness occurring in the first loft. No further reports were received after late June 2016.

In October 2016 similar cases were reported in a small town approximately 200km south of Perth. As with the previous cases, affected birds had reportedly shared transportation with birds from the Perth flocks that developed disease. High morbidity and mortality were recorded but no disease was reported in Perth lofts at this time, nor in feral or fancy birds.

In December 2016, similar signs were reported in Victoria following a sale that had been attended by Western Australian fanciers. Reports indicated that affected birds usually died within 12 to 24 hours from onset of vomiting, with deaths continuing for about seven days. Between 15 and 45% of birds in affected lofts died, mostly young birds. By early 2017 cases had been identified in all Australian states apart from the Northern Territory, (S1 Fig) and in all classes of domestic pigeons, including fancy birds and meat pigeons; racing pigeons, however, continued to be the most commonly affected group. In April 2017 mild enteric disease of low mortality rate (approximately 2%) was reported in some previously unaffected lofts in southeastern Queensland. No further reports of feral pigeon die off or of disease in native pigeons were received during this period.

### Gross and microscopic pathology

**Fig 1:**
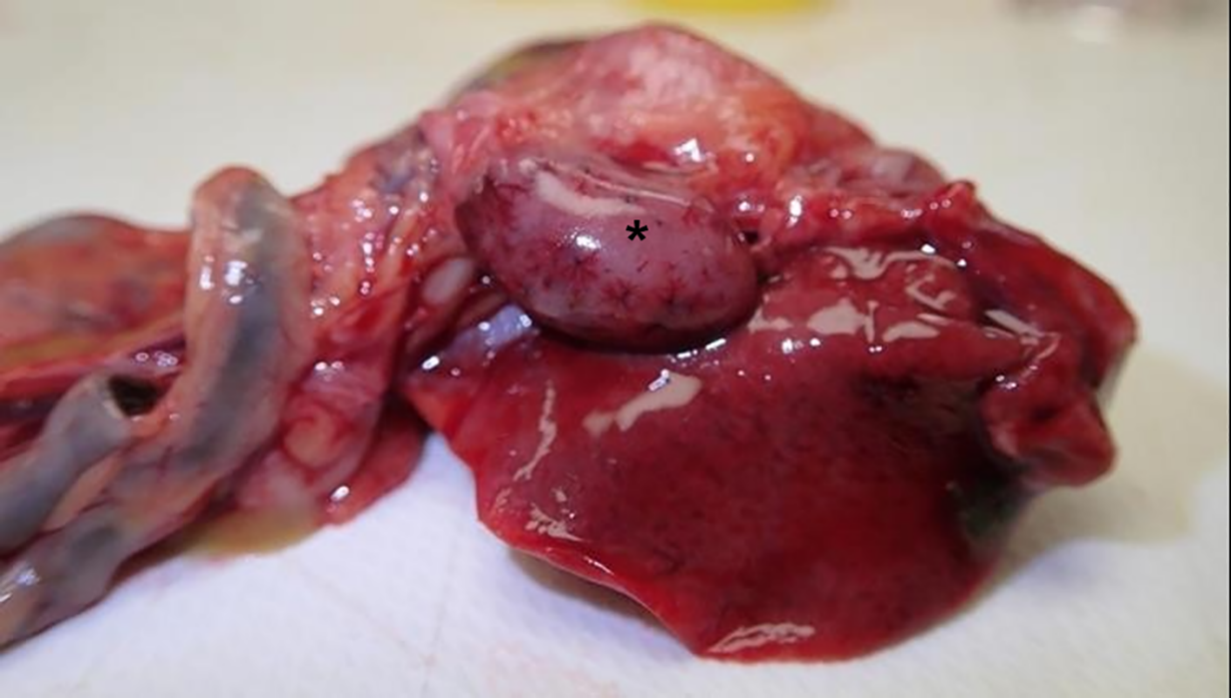
Enlargement of the spleen (*) with mild haemorrhage was seen in some early cases.

Gross lesions were minor; no lesion was consistently present in all birds, and many birds had no convincing macroscopic changes. Soiling of the vent and presence of ingesta on the beak, suggestive of diarrhoea and vomiting, were sometimes seen, but not in all cases. Western Australian autopsies showed mildly enlarged, diffusely mottled or congested livers and enlarged, friable and mottled spleens (Fig 1). Subsequent autopsies in Victoria showed only subjective friability of the liver, particularly in the birds that had been euthanased or had died en route (ie not dead for more than a few hours) and possible mild pallor. This was not evident in birds dead for longer periods. Spleens were rarely notable, although mild enlargement was occasionally seen. Renal pallor was noted in up to 25% of birds, without urate distension of ureters.

**Fig 2:**
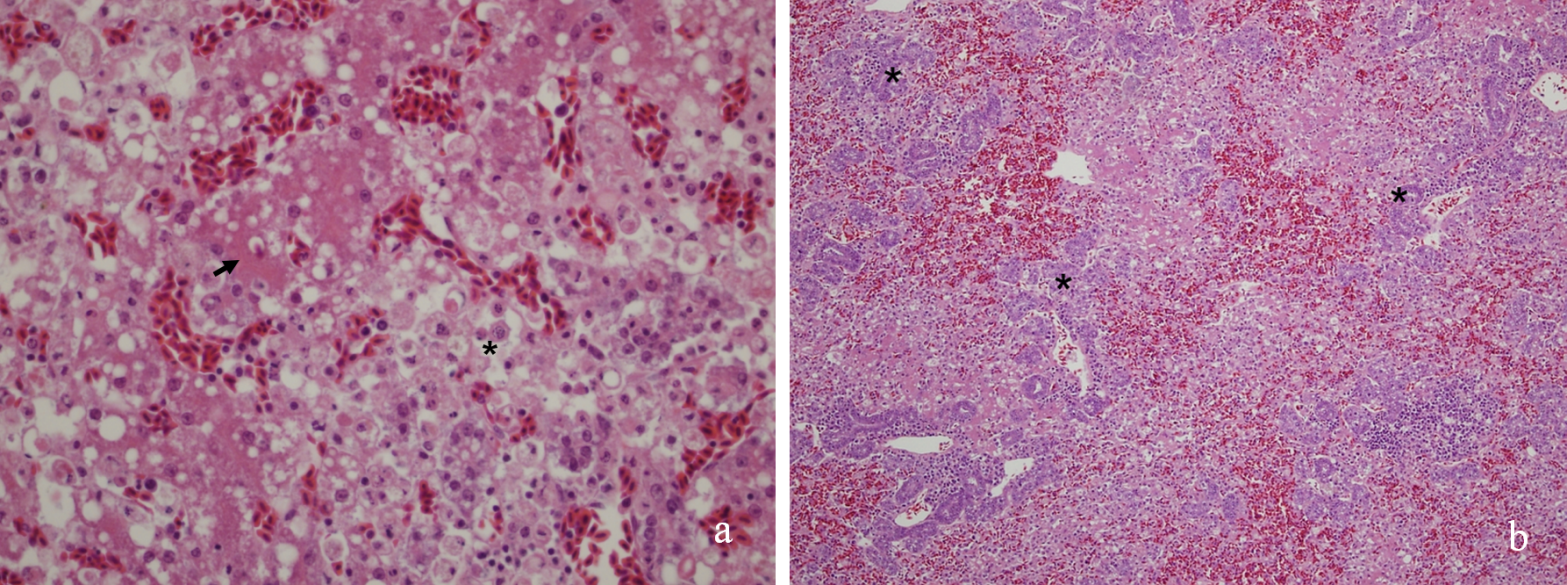
a) Hepatocellular dissociation with necrosis and macrophage infiltrate (*), in the absence of other inflammatory response, was the distinctive finding in all birds, although severity varied. Islands of relatively normal parenchyma (arrow) remained. (b) Biliary proliferation (*) was sometimes seen.

Histologically, the liver was targeted in all birds, and showed variably severe hepatocellular dissociation and necrosis (Fig 2A), often following irregular serpiginous paths in the parenchyma, or occasionally with mild periacinar sparing. Some birds had prominent biliary proliferation (Fig 2B). In most livers there was a macrophage infiltrate, but often little other evidence of inflammation or response. In many Western Australian birds, distinctive amorphous eosinophilic cytoplasmic inclusions (presumed cytosegresomes) with otherwise clear cytoplasm and a crescent shaped marginated nucleus (Fig 2A) were seen in occasional cells within the necrotic zones. These were not present in the birds from the eastern states.

The spleen usually also showed lesions but these were inconsistent, with the commonest change being histiocytosis. Macrophages from the Western Australian birds contained similar intracytoplasmic inclusions, as in the liver not seen in eastern birds. Lymphoplasmacytic infiltrates were sometimes seen, with the histiocytosis or as a main lesion and there was frequently widespread apoptosis of parenchymal cells. No consistent lesions were seen in other organs.

Occasional birds showed circoviral inclusions in lymphoid tissues. Some Victorian submitters sent samples to an external provider for circovirus PCR; results indicated that up to 50% of birds were infected.

### Pathogen screening

Two liver samples yielded heavy growth of *Escherichia coli*, but livers from two other birds from the same loft gave no growth. Overall, 16 liver samples and 3 faecal samples were negative on routine culture or showed light mixed growth considered to be contamination of the sample. As well as the two heavy cultures of *E*.*coli* there were two deemed to be moderate for this organism. No sample was positive for *Salmonella* sp.

All tested samples were negative for *Chlamydia*, herpesvirus and adenovirus DNA.

### Electron microscopy

**Fig 3:**
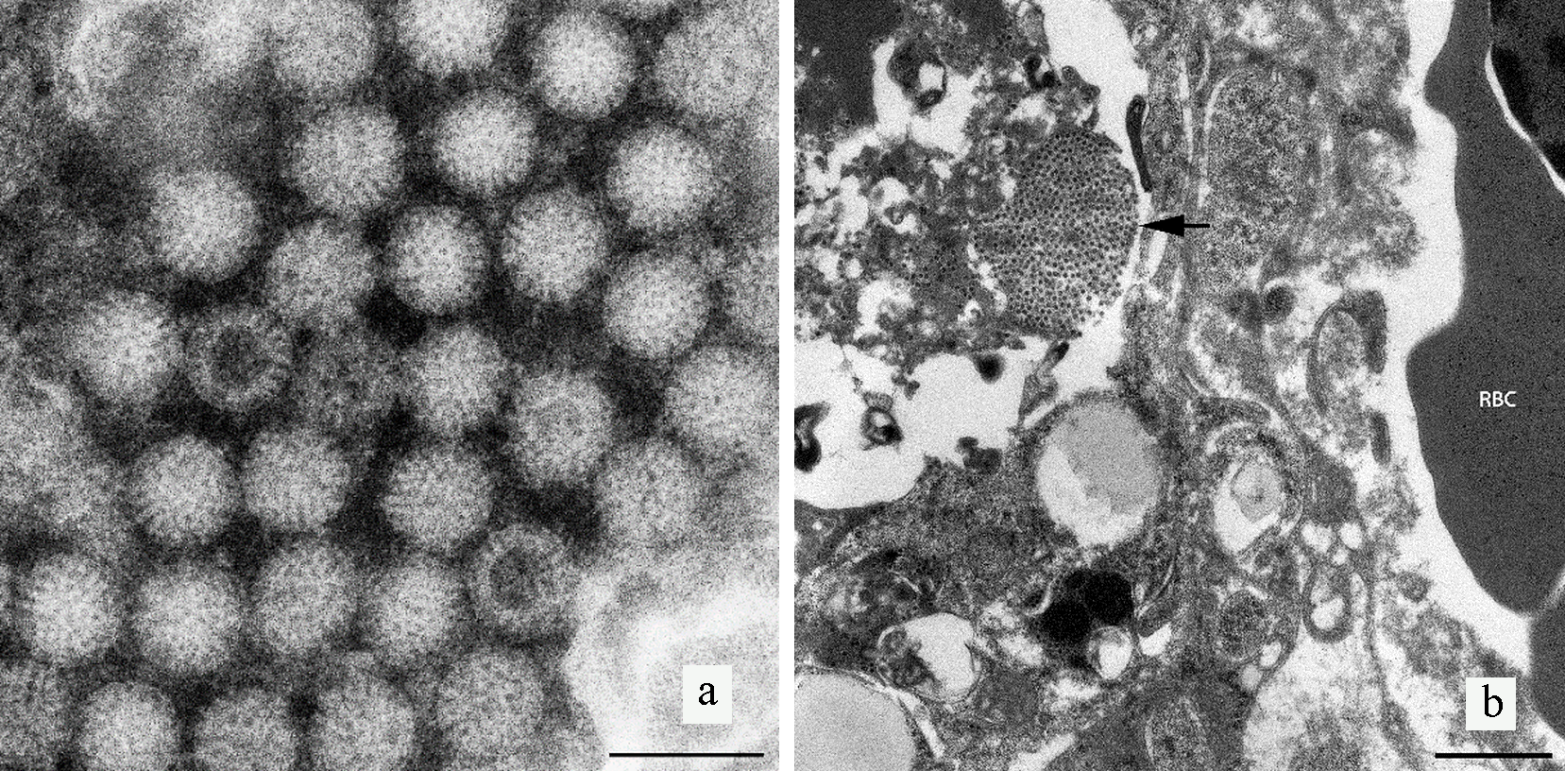
a) Negative contrast electron microscopy revealed non- enveloped viral particles. Viral particles (arrows) have electron dense cores, where stain has penetrated the interior of the particles, clearly demonstrating a wheel-like appearance consistent with the family *Reoviridae*, genus *Rotavirus*. Scalebar represents 100nm. b) Ultrathin section of infected pigeon liver tissue. Arrow indicates an array or cluster of virus particles egressing from a dying cell. RBC = Red blood cell. Scale bar represents 1μm.

Examination of negative contrast preparations of liver via TEM revealed virus particles resembling those of the family *Reoviridae* (Fig 3A). The virions were non-enveloped, with wheel-like layers projecting from an electron dense core. The particles possessed a mean diameter of 72.8 ± 1.72 nm (n = 50). In ultra-thin sections, viruses were observed in the cytoplasm in arrays or as individual particles (Fig 3B).

### Virus isolation

#### Virus isolation in cell lines

**Fig 4:**
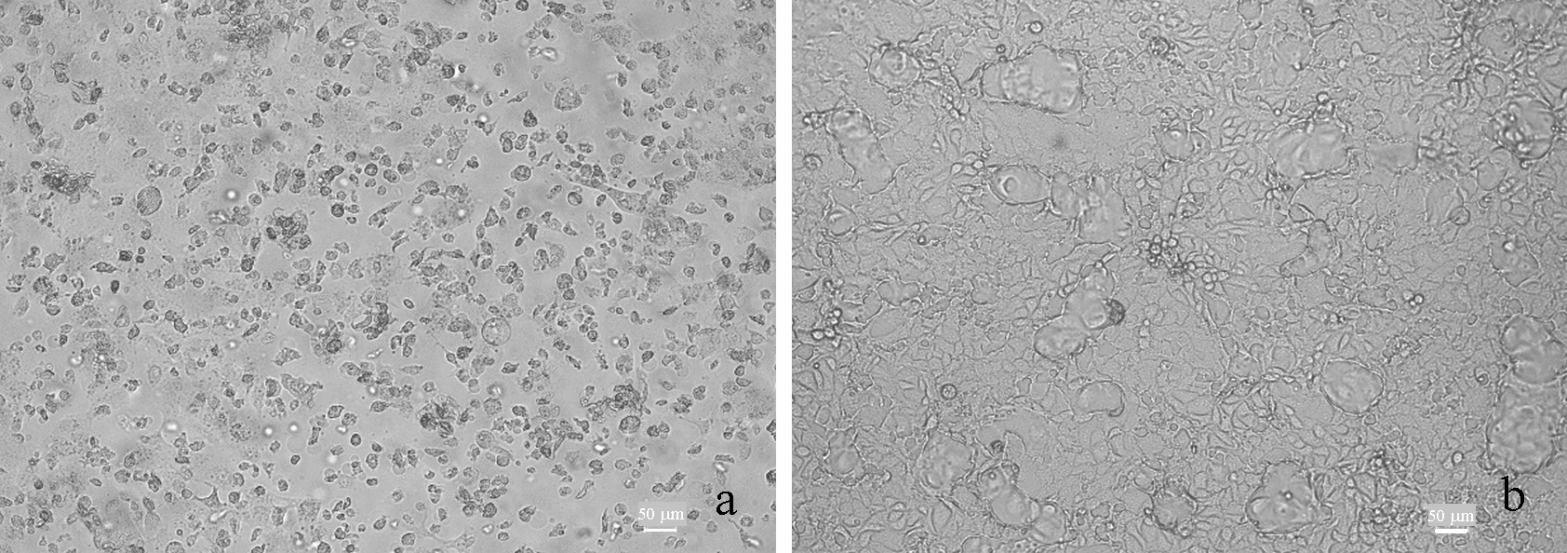
Cytopathic effect induced by pigeon rotavirus in MA-104 cells, three days post-infection. (A) Uninfected control cells. (B) Infected cells.

The novel group A rotavirus (RVA) was isolated using MA104. Reliable CPE was was characterised by round or spindle-shaped cells, cellular granulation and eventual cell detachment from the vessel (Fig 4)The presence of RVA in MA-104 cells was further confirmed by IFA with RVA-specific antibodies. Ct values indicated higher viral loads in MA104 cells and generally decreased in MA104 cells over three passages and viral particles consistent with rotaviruses were seen by negative contrast EM of infected MA-104 cells (S2 Fig). CPE was seen in Vero and MDBK in one laboratory,with stationary Ct values (allowing for dilution) indicating isolation without amplification, but was unreliable in other hands. CPE was absent or unreliable in other cell lines.

### NGS analysis

NGS analysis of a sample from a pigeon with severe hepatic necrosis from the second loft to submit samples in Victoria identified the complete genome sequence of a novel rotavirus including all 11 genome segments. Subsequently all high pathogenicity outbreaks were confirmed by sequence comparison to be associated with the same virus (manuscript in preparation). Swabs from the low pathogenicity outbreak in Queensland were positive on screening PCR, but had insufficient nucleic acid for sequencing.

Phylogenetic analysis grouped the virus into the avian subgroup of RVA and identified the genotype as G18P[17] based upon the VP7 and VP4 sequences. The genomic constellation of all 11 genome segments was classified as G18P[17]-R4-C4-M4-A4-I4-T4-N4-E19-H4. The complete sequences of each individual genes were submitted to GenBank, Accession No. MH668302-MH668312.

**Fig 5:**
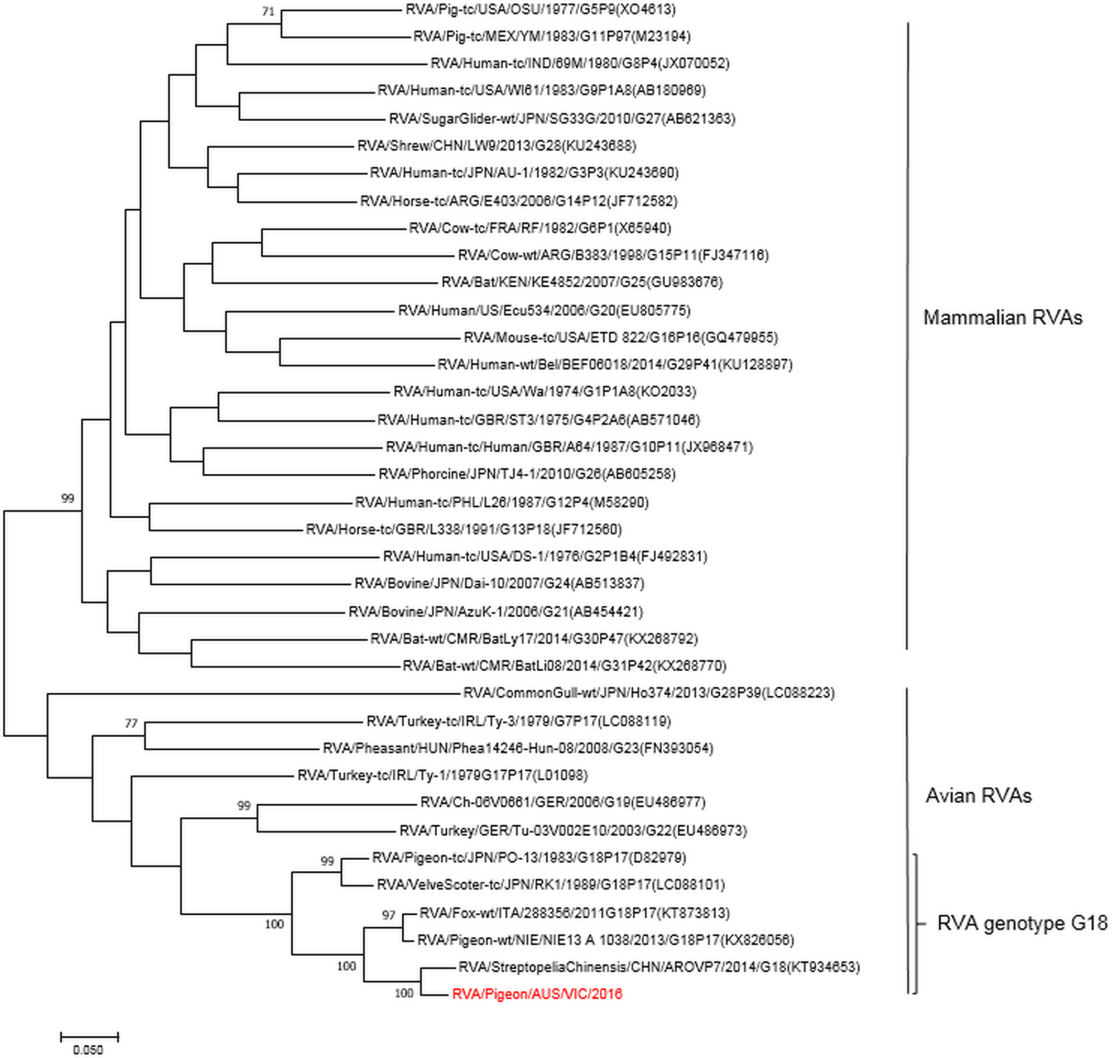
Phylogram indicating genetic relationships of complete nucleotide sequences of VP7 of pigeon rotavirus strain RVA /pigeon-wt/AUS/VIC/2016/G18P[17] (in red) from Australia with representatives of known human and animal rotavirus genotypes. The tree was generated by the Maximum Likelihood method using MEGA7. Bootstrap values (1000) above 70 are shown. Scale bar indicates nucleotide substitutions per site.

Sequence analysis showed a high sequence identity of the pigeon isolate to a rotavirus virus strain fox-wt/ITA/288356/2011/G18P[17] isolated from a red fox, and avian rotavirus strains AROVP1 (KT934648) isolated from a spotted dove and RVA/pigeon-tc/JPN/PO-13/1983/G18P[17] from a healthy feral pigeon (Fig 5). The NSP4 segment belongs to the E19 genotype first described in this fox [20] and subsequently reported from avian samples in Nigeria [13].

The comparison of individual gene segments of the rotavirus from Victoria with the fox isolate and pigeon rotavirus PO-13 is summarised in Table 2.

**Table 2:** Comparison of gene segments of the rotavirus from Victoria with RVA isolates fox-288356 and PO-13.

Gene-coding Segments	Fox-288356 NA Identity	PO-13 NA Identity
**VP1**	97.2%	92.8%
**VP2**	94.6%	92.1%
**VP3**	96.0%	90.2%
**VP4**	94.7%	90.9%
**VP6**	94.4%	92.1%
**VP7**	90.7%	85.3%
**NSP1**	94.2%	85.6%
**NSP2**	94.2%	92.7%
**NSP3**	96.4%	88.4%
**NSP4**	96.4%	76.3%
**NSP5**	97.5%	94.6%

### PCR testing

PCR and sample validation values are shown in S1 and S2 Tables. All sample types showed amplification with all primer sets, confirming the utility of cloacal swabs for screening of live birds. All histologically confirmed positive samples and all samples from clinically suspicious birds sampled early in the outbreak produced PCR product, as did samples from Queensland birds from the low pathogenicity episode. Swabs from apparently recovered birds were also positive, and shedding of viral RNA from some apparently healthy, recovered birds continued for at least 10 weeks after cessation of diarrhoea and vomiting.

The PCR assay targeting the VP6 gene segment proved to be the most sensitive qPCR assay producing lower cycle of threshold (Ct) values for equivalent samples in comparison to the qPCR assays based upon the VP7 and NSP3 gene segments. Three birds with positive histological diagnosis and positive results using VP6 were negative for VP7, and one was also negative using NSP3 primers. Two samples with positive Ct values using NSP3 were negative for VP6 and VP7. Histological confirmation of the results was not available.

## Discussion

We have isolated a previously unidentified rotavirus from livers of pigeons suffering from diarrhoea and hepatic necrosis. The initial investigations in Western Australia did not include healthy birds, but at the onset of disease in Victoria, both clinically affected and unaffected birds, and archival samples, were tested and only clinically affected pigeons were found to harbour the virus suggesting a link between the virus and the disease. This is supported by the failure to detect other pathogens by histological examination, routine bacterial culture or virus isolation, or to detect other agents in lesioned tissue by EM. Metagenomic analysis using NGS on RNA extracted from lesioned tissue did not find genetic evidence of other potential pathogens.

The virus and associated disease progressively occurred throughout mainland Australia and Tasmania over a period of about eleven months. It is likely that racing pigeon movements contributed largely to this spread (Hunnam et al, manuscript submitted), but in some cases links to previously infected lofts were not identified. RVA RNA was detectable by PCR from recovered birds for several months following cessation of vomiting and diarrhoea, but it is not known if active infection can be spread this way, and the role of feral or native birds in virus spread is unknown. Following the introduction of pigeon paramyxovirus to Victoria in 2011, obvious severe reduction in flock size of feral pigeons was seen (unpublished observations), but no similar effect was noted following the outbreaks of rotavirus associated disease, and only one episode of significant mortalities in feral pigeons was reported. Rotaviruses can persist on fomites and in water for extended periods of time [39–41], offering other routes of translocation, and transmission via exposure to transport vehicles was likely in some instances.

Avian RVAs generally fall into a separate genotype and show different electrophoretic patterns from mammalian viruses [42], suggesting that transmission of avian viruses to mammals should be unlikely. However, sequence comparisons indicate a close relationship between the virus found in Australian pigeons and a rotavirus recently isolated from the brain of a fox with encephalitis, in Italy [20]. The authors reported their isolate to be evolutionarily close to a rotavirus strain, PO-13, which was originally isolated from a Japanese feral pigeon [17] and was the first full length avian rotavirus genome published [18]. Of the avian rotaviruses tested, only PO-13 has shown the capacity to infect mice experimentally [19], and reports of avian group viruses isolated from mammals show a close relationship with PO-13 [20, 22].

The fox isolate contained a novel NSP4 region, designated E19 [20] which is also present in our isolate. NSP4 is not usually conserved between mammals and birds, with homology calculated at 32–35% for PO-13 and selected mammalian viruses [18]. Although first described in a mammalian isolate, investigation of birds in farms and live bird markets in Nigeria suggest that G18-P[17]-[I4]-E19 may be a common genotype of pigeon RVAs [13], further supporting an avian origin for this isolate. The potential for the virus described here to cross species barriers is unknown; to date no disease has been recognised in species other than *Columba livia*, but further investigation of its cross- species pathogenicity may be warranted.

The pathogenesis of rotaviral disease is incompletely understood [27], even for intestinal disease. However, extra-intestinal disease associated with rotavirus infection is becoming increasingly recognised [20, 27, 33, 43], with liver, CNS and respiratory systems particularly, but not exclusively, reported as the targets of disease [27]. Abnormalities in liver function tests and transient hepatitis are seen in association with gastrointestinal rotaviral disease [27, 44]. Viraemia is seen more commonly than overt disease, raising the question of whether the viral incursion is causative or secondary in pathogenesis [27–29, 31, 32, 45].

In immunodeficient children with multiple infections that include systemic rotavirus, the liver and kidney may be the major, or only, extraintestinal sites of virus recovery [43] and in SCID mice infected with a rhesus rotavirus, virus was demonstrated only in liver and intestine [46], suggesting that the liver may be particularly supportive of rotaviral gene expression. Rotaviruses have also been isolated from lung in pneumonias [47–49], brain in cases of encephalitis [50–52] and from other sites, such as myocardium [47].

Immunocompromise, be it due to age, secondary to other disease or congenital, is regarded as a risk factor for intestinal rotaviral disease, but its role in extraintestinal disease is less clear [27]. Experimental infection of mice with a rhesus rotavirus resulted in higher rates of hepatic lesion development and death in SCID animals, compared with those having normal immune systems; in neither group did infection with a murine or bovine rotavirus lead to extraintestinal spread [46]. However, experiments with rats, using the same rhesus virus, showed that neither active nor passive immunity was protective [53].

The pigeons reported in this case were of various ages, including adults, and no concurrent disease was identified. Histologically, there was no indication of splenic or bursal depletion, where the tissue was available, although inclusion bodies typical of circovirus were seen in two birds and pigeon circovirus was reportedly present in about half the birds (n = approximately 17 birds tested) tested by PCR for that agent. Circoviral infection is widespread and common in racing pigeons [54] and it has been associated with disease susceptibility in young pigeons [54–56]. The inconsistent finding of circovirus infection and the wide range of ages of birds affected make the role of circovirus uncertain in our cases. No other reason for hepatic disease was identified in the pigeons submitted, but we have not undertaken experimental infections to confirm that the lesions seen will occur in the absence of other disease.

Viral factors promoting extraintestinal spread are not understood. NSP4 and its secreted fragment have been shown to have an enterotoxic activity, which can provide a paracrine mechanism to mediate diarrhoea in the absence of significant histological changes [27, 57–60], and this has been proposed as a mechanism of extra-intestinal pathogenesis [61]. It is interesting that the NSP4 sequence of the virus isolated from the pigeons was closely related to that from a virus isolated from lesioned brain in a fox.

Approximately ten months after the first cases were reported, further episodes of high mortality disease associated with rotavirus shedding in faeces occurred first in Western Australia then in the eastern states, again affecting birds of various ages, including birds that were reported to have recovered from a previous disease episode. Tissues from the Western Australian birds had consistent histological lesions and were positive for pigeon rotavirus PCR. In the eastern states, lesions were inconsistent, although positive results were returned from swabs.

Reinfection with rotaviruses following recovery from enteritis is reported in mammalian species [45, 62]. Subsequent infections generally induce milder or no clinical signs, and immunity may not be long lasting [45]. Data on avian species are sparse; sequential infection of flocks with different rotavirus serogroups has been documented, but disease status of the birds was not recorded [9]. Recurrent infection with high mortality is unusual; anecdotally adult birds were among those that died in the repeated disease episodes and mortality rates in recurrently infected lofts were reported to be high, as in the initial disease outbreak.

During the initial spread of virus around the country there was a local episode of disease of apparently lower virulence. Swabs from affected birds were positive for the new strain by PCR and histological appearance of liver in birds which died was consistent with that of the birds dying in the main outbreak, but mortality rates were low. It is possible that this was a result of environmental or management factors as virus loads in these mildly affected birds were comparatively low. However, rotaviruses show a high degree of genetic diversity, with isolates even varying within an outbreak or within individuals in an outbreak; it has been suggested that the comparison between a rotavirus and its parent genome will frequently show at least one offspring mutation [63]. Rotaviruses also show frequent random reassortment of genome segments, often giving phenotypic changes [63], so a lower virulence strain is also possible, but we have not yet been able to fully sequence isolates from these birds and strain variation has not been demonstrated.

Further investigation is needed to identify the origin of this virus. Closely related virus sequences have been identified in tissues from pigeons in Germany and other European countries (Rubbenstroth et.al., manuscript submitted) and the Italian fox isolate remains the closest published sequence. The robust nature of rotaviruses and the European origin of the closest evolutionary neighbours currently identified, suggests a possible external introduction, perhaps with subsequent modification leading to increased virulence when the virus arrived in Australia. Sequence comparison between viruses from the initial outbreaks, subsequent recurring episodes and the lower mortality disease episodes, and with pigeon viruses from Europe, may allow better understanding of the evolution of this agent and may also improve understanding of factors associated with extra-enteric rotaviral disease. Koch’s postulates remain to be fulfilled by challenge trials, testing the role of other agents such as circovirus in the pathogenesis of rotavirus associated hepatic necrosis.

## Supporting information

S1 Fig Map of Australia showing the progression of virus detection across the country. (TIF)

S2 Fig Transmission electron micrograph of virus obtained from the third passage of pigeon rotavirus in MA-104 cells (A) Particles with a wheel-like appearance as seen by Negative contrast EM. Bar, 100 nm. (B) Arrows indicates replicating virus particles in the rough endoplasmic reticulum. N = nucleus, C = cytoplasm, ECS = extracellular space. Bar, 1um.
(TIF)

S1 Table Samples tested to validate rotavirus primers and probes. (DOCX)

S2 Table Ct values comparing sensitivity of different primer sets for rotaviral detection. Samples labelled “Negative” showed no amplification at 40 cycles. Samples listed as “Swab in VTM” were not submitted without identification of site. VTM–virus transport medium.
(DOCX)
