# Supplementary figures and images for: A novel group A rotavirus associated with acute illness and hepatic necrosis in pigeons (Columba livia), in Australia
Source: PLoS One. 2018 Sep 11;13(9):e0203853. doi: 10.1371/journal.pone.0203853 (PMC6133385; doi:10.1371/journal.pone.0203853)

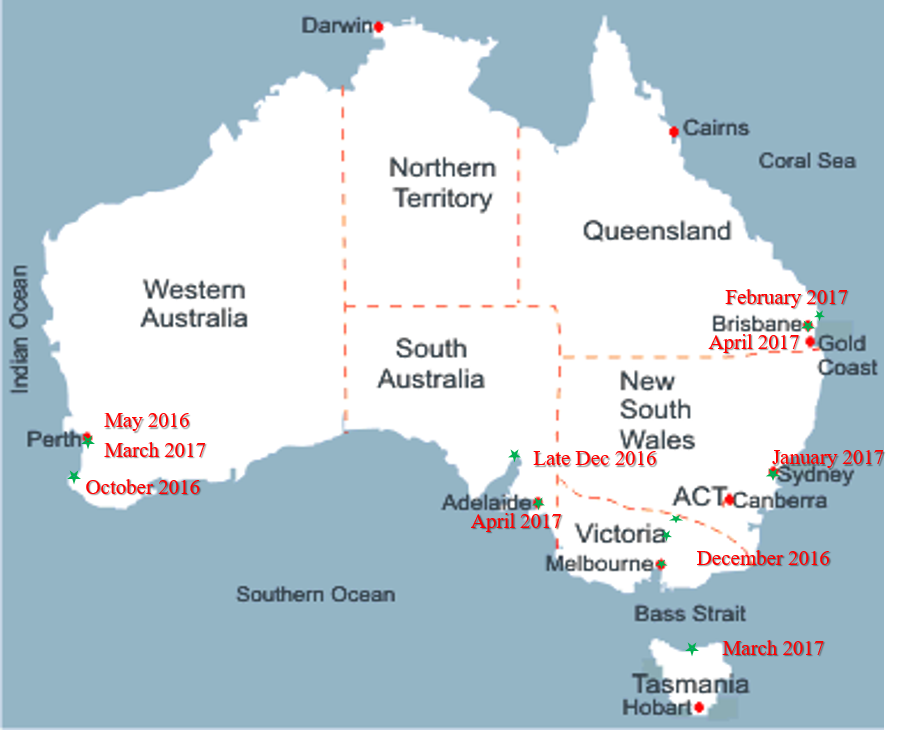

Supplement: S1 Fig — (TIF) [file pone.0203853.s001.tif]

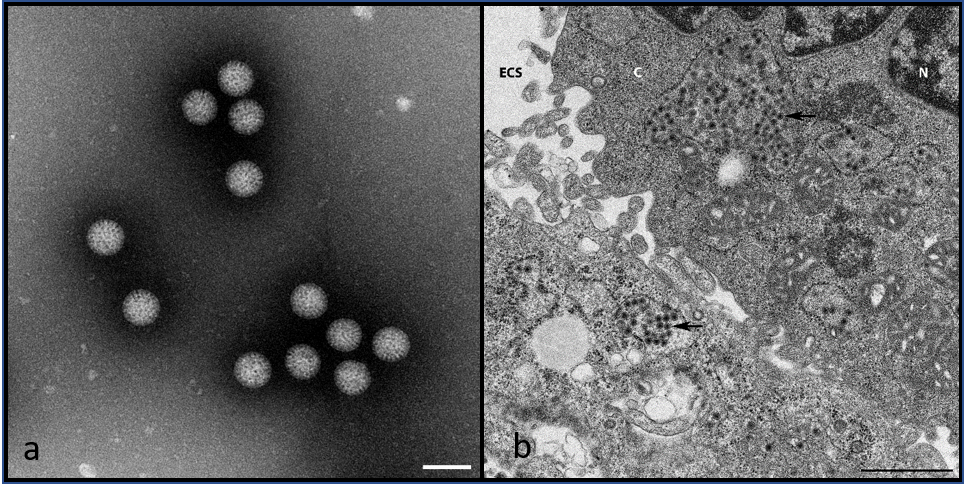

Supplement: S2 Fig — Transmission electron micrograph of virus obtained from the third passage of pigeon rotavirus in MA-104 cells (A) Particles with a wheel-like appearance as seen by Negative contrast EM. Bar, 100 nm. (B) Arrows indicates replicating virus particles in the rough endoplasmic reticulum. N = nucleus, C = cytoplasm, ECS = extracellular space. Bar, 1um. (TIF) [file pone.0203853.s002.tif]
