# Supplementary material for: A novel group A rotavirus associated with acute illness and hepatic necrosis in pigeons (Columba livia), in Australia
Source: PLoS One. 2018 Sep 11;13(9):e0203853. doi: 10.1371/journal.pone.0203853 (PMC6133385; doi:10.1371/journal.pone.0203853)
Supplement: S2 Table — Samples labelled “Negative” showed no amplification at 40 cycles. Samples listed as “Swab in VTM” were not submitted without identification of site. VTM–virus transport medium. (DOCX) [file pone.0203853.s004.docx]

| **SAMPLE TYPE** | **VP-6** | **VP-7** | **NSP3** | **Comments** |
| --- | --- | --- | --- | --- |
|  |  |  |  | **Histologically confirmed to have hepatic necrosis** |
| Cells from virus isolation | 26.67 | 27.93 | 27.29 |  |
| Swab in VTM | 19.96 | 14.08 | 22.66 |  |
| Swab in VTM | 24.61 | 20.86 | 28.49 |  |
| Swab in VTM | 23.05 | 23.82 | 23.64 |  |
| Swab in VTM | 25.09 | 26.03 | 25.72 |  |
| Cloacal swab | 21.78 | 22.36 | 22.14 |  |
| Swab in 20mL saline | 28.22 | 29.15 | 30.20 |  |
| Cloacal swab | 18.22 | 18.69 | 18.63 |  |
| Cloacal swab | 21.26 | 22.10 | 22.02 |  |
| Liver swab | 19.16 | 20.72 | 18.95 |  |
|  |  |  |  | **Histologically confirmed to have no hepatic necrosis** |
| Cloacal & Choanal swab | Negative | Negative | Negative |  |
| Liver tissue | Negative | Negative | Negative |  |
| Cloacal swab | Negative | Negative | Negative | feral pigeon |
| Liver tissue | Negative | Negative | Negative | native pigeon |
|  | Negative | Negative | Negative | native pigeon |
|  |  |  |  | **No histological examination: clinical disease** |
| Swab in VTM | 25.35 | 25.86 | 27.36 |  |
| Swab in VTM | 29.20 | 30.60 | 30.80 |  |
| Swab in VTM | 34.05 | 35.76 | 35.25 |  |
| Swab in VTM | 36.14 | 35.54 | 35.57 | Qld |
| Swab in VTM | 31.86 | 33.39 | 32.65 | Qld |
| Swab in VTM | 35.06 | Negative | 38.45 |  |
| Swab in VTM | 31.94 | 32.77 | 32.24 |  |
| Swab in VTM | 34.36 | 42.59 | 35.15 |  |
| Swab in VTM | 31.47 | 31.76 | 31.21 |  |
| Swab in VTM | 35.94 | Negative | 36.97 |  |
| Swab in VTM | 33.02 | 33.62 | 33.45 |  |
| Swab in VTM | 33.71 | 35.24 | 34.74 |  |
| Swab in VTM | 33.07 | 34.38 | 34.98 |  |
| Swab in VTM | 29.00 | 31.02 | 32.65 |  |
| Swab in VTM | 34.05 | 35.28 | 35.11 |  |
| Swab in VTM | 34.09 | 36.13 | 35.74 |  |
| Swab in VTM | 34.65 | 35.73 | 35.39 |  |
| Swab in VTM | 34.97 | 38.64 | 36.81 |  |
| Swab in VTM | 34.76 | 35.85 | 36.69 |  |
| Swab in VTM | Negative | Negative | 38.09 |  |
| Swab in VTM | 34.03 | 36.00 | 34.61 |  |
| Swab in VTM | 35.57 | 35.19 | 37.33 |  |
|  |  |  |  | **No histological examination: recovered from clinical disease** |
| Swabs in VTM | 29.10 | 31.14 | 31.33 | Recovered birds - 4 weeks |
|  | 38.64 | Negative | Negative |  |
|  | 32.22 | 33.4 | 34.08 |  |
|  | 31.75 | 33.06 | 32.93 |  |
| Cloacal swabs | 28.78 | 29.38 | 29.40 | Recovered birds - 7 weeks |
|  | 35.69 | 35.38 | 36.12 |  |
|  | 31.32 | 31.94 | 32.62 |  |
|  | 26.27 | 27.65 | 27.41 |  |
|  | 32.73 | 33.39 | 34.12 |  |
|  | 32.32 | 33.01 | 33.15 |  |
|  | 32.35 | 33.32 | 33.72 |  |
|  | 33.87 | 33.70 | 34.73 |  |
|  | 29.27 | 30.27 | 30.30 |  |
|  | 32.48 | 33.23 | 32.83 |  |
| Swabs in VTM | 37.18 | Negative | 37.00 | Recovered birds - 10 weeks |
|  | Negative | Negative | 38.38 |  |
|  | 33.29 | 33.79 | 34.17 |  |
|  | 36.46 | Negative | Negative |  |
|  | 35.9 | 39.71 | 38.06 |  |
|  | Negative | Negative | Negative |  |
|  | Negative | Negative | Negative |  |
|  | Negative | Negative | Negative |  |
|  | 37.78 | 37.15 | 37.43 |  |
|  | Negative | Negative | Negative |  |
|  | 33.4 | 34.06 | 34.22 |  |
|  |  |  |  | **No histological investigation; no clinical disease reported** |
| 20 Choanal Swabs | All neg | All neg | All neg | 2 different lofts, 10 birds each |
| 20 Cloacal swabs | All neg | All neg | All neg | Same lofts as above |
| 10 Swabs in VTM | Negative | Negative | Negative | 10 separate lofts |
| 4 Dry Swabs | Negative | Negative | Negative |  |
| Swab in VTM | Negative | Negative | Negative | Not a pigeon – silver gull |
| Negative cell culture control | Negative | Negative | Negative |  |
