# Supplementary material for: A novel group A rotavirus associated with acute illness and hepatic necrosis in pigeons (Columba livia), in Australia
Source: PLoS One. 2018 Sep 11;13(9):e0203853. doi: 10.1371/journal.pone.0203853 (PMC6133385; doi:10.1371/journal.pone.0203853)
Supplement: Supplementary file 3 [file pone.0203853.s003.docx]

Sample type	VP-6	VP-7	NSP3
L1 liver	9.3	10.61	10.18
L1 homogenate	8.23	9.77	9.61
L1 liver Bird 3	13.97	14.89	13.55
(L1) Vero_RTT P2 neat	26.25	27.28	26.95
(L1) Vero_RTT P2 1:10	29.04	30.15	29.99
(L1) Vero_RTT P2 1:100	31.8	32.92	32.63
(L1) Vero_RTT P2 1:1000	35.67	34.95	35.37
CMDBK TT 1:10	23.77	24.52	24.42
CMDBK RTT neat	26.18	26.97	26.7
CMDBK RTT 1:10	26.69	27.42	27.24
CMDBK RTT 1:100	31	31.71	31.54
Vero TT neat	18.58	19.73	19.51
Tissue 4 VTM	12.24	13.47	13.26
2) P2 Vero UT 1:10	24.22	24.97	24.95
5) P2 Vero TT 1:11	26.23	27.33	27.13
2) P2 CMDBK UT 1:10	22.77	23.82	23.71
11) P2 CMDBK RTT 1:1000	34.29	35.43	35.6
AF P2 16/2/17	Negative	Negative	Negative
General Po VISO	26.67	27.93	27.29
